# 中国肺部结节分类、诊断与治疗指南(2016年版)

**DOI:** 10.3779/j.issn.1009-3419.2016.12.12

**Published:** 2016-12-20

**Authors:** 清华 周, 亚光 范, 颖 王, 友林 乔, 贵齐 王, 云超 黄, 新允 王, 宁 吴, 国桢 张, 向鹏 郑, 宏 步

**Affiliations:** 1 610041 成都，四川大学华西医院肺癌中心 Lung Cancer Center, West China University, Sichuan University, Chengdu 610041, China; 2 300052 天津，天津医科大学总医院，天津市肺癌研究所 Tianjin Lung Cancer Institute, Tianjin Medical University General Hospital, Tianjin 300052, China; 3 100021 北京，中国肺癌早诊早治专家组 China National Expert Group of Early Diagnosis and Treatment of lung Cancer, Beijing 100021, China; 4 300052 天津，天津医科大学总医院 Tianjin Medical University General Hospital, Tianjin 300052, China; 5 100021 北京，国家癌症中心/中国医学科学院肿瘤医院 National Cancer Center/Cancer Hospital, Chinese Academy of Medical Sciences, Beijing 100021, China; 6 650105 昆明，昆明医科大学附属肿瘤医院 Cancer Hospital, Kunming Medical University, Kunming 650105, China; 7 200040 上海，上海华东医院放射科 Department of Radiology, Shanghai Huadong Hospital, Shanghai 200040, China; 8 610041 成都，四川大学华西医院病理科 Department of Pathology, West China Hospital, Sichuan University, Chengdu 610041, China

肺癌是我国最常见的恶性肿瘤之一，其死亡率无论是在城市或乡村、男性或女性，均居癌症死亡的首位^[1]^。由于绝大多数临床诊断肺癌病例多已为晚期，失去手术治疗机会，肺癌预后极差，我国肺癌的5年生存率仅为16.1%^[2]^。因此，肺癌的筛查是改善肺癌生存，降低肺癌死亡率的希望所在。2011年，美国国家肺癌筛查试验(National Lung Screening Trial, NLST)首次报告低剂量螺旋计算机断层扫描(low-dose computed tomography, LDCT)筛查在高危人群中可显著降低肺癌的死亡率^[3]^。基于此结果，多家医学机构已建议在肺癌高危人群中开展低剂量螺旋CT筛查^[4-7]^。鉴于肺癌的疾病负担，我国自2009年起开始启动了农村肺癌早诊早治项目，在项目点高危人群中开展LDCT筛查，显著提高了当地肺癌的早期检出率，并由此根据项目技术方案制定了我国肺癌筛查指南^[8, 9]^。然而，临床CT检查及LDCT筛查中，大量假阳性结节的检出仍是亟需解决的一个问题，在NLST研究中，CT筛查组中96.4%的阳性结节为良性，我国农村肺癌早诊早治项目的假阳性率也较高。过高的假阳性可能导致过度诊断、过度治疗、医疗资源的浪费及增加受检者焦虑心理^[10]^。因此，有效地对肺部结节进行鉴别诊断，快速明确其良恶性，尽早切除恶性结节，同时避免不必要的过度治疗，是肺部结节诊断治疗的关键，国外多个组织也制定了肺部结节处理指南^[11-15]^。中国肺癌早诊早治专家组结合国内外最新的肺结节处理指南及在我国临床及人群筛查的实践，经过充分讨论，制定了《中国肺部结节分类、诊断与治疗指南(2016年版)》。

## 1 肺结节的定义及分类

肺结节(pulmonary nodule, PN)是指肺内直径小于或等于3 cm的类圆形或不规则形病灶，影像学表现为密度增高的阴影，可单发或多发，边界清晰或不清晰的病灶。不同密度的肺结节，其恶性概率不同，依据结节密度将肺结节分为三类：实性结节(solid nodule)、部分实性结节(part-solid nodule)和磨玻璃密度结节(ground glass nodule, GGN)。其中，部分实性结节的恶性概率最高，依次为磨玻璃密度结节及实性结节。磨玻璃密度结节是指肺内模糊的结节影，结节密度较周围肺实质略增加，但其内血管及支气管的轮廓尚可见。实性结节是指其内全部是软组织密度的结节，密度较均匀，其内血管及支气管影像被掩盖。部分实性结节是指其内既包含磨玻璃密度又包含实性软组织密度的结节，密度不均匀(图 1)。

**1:**
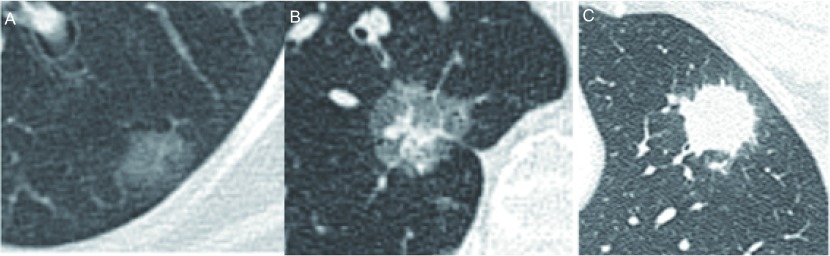
肺结节的密度分类。A：磨玻璃结节；B：部分实性结节；C：实性结节。 Classification of lung nodule density. A: Ground-glass nodule; B: Part solid nodule; C: Solid nodule.

## 2 肺结节的评估方法

肺结节的评估方法主要包括个体或临床特征、影像学方法和临床肺癌概率。

### 一 临床评估

临床评估包括患者的病史和体征检查，包括年龄、性别、职业、吸烟史、慢性肺部疾病史、个人和肿瘤家族史、职业暴露史等。临床的信息可为肺部结节的鉴别诊断提供参考依据。

### 二 影像学技术

胸部X光片、CT及磁共振成像(magnetic resonance imaging, MRI)均可以检测到肺结节，但鉴于胸部CT的高空间分辨率及成像方便快捷的优势，应以胸部CT检查作为肺结节的标准检查方法。对胸部不定性结节常需要进行多次随访，建议采用低剂量扫描技术以降低放射损伤。建议为：(1)采用螺旋CT容积扫描技术，依据受试者体重，管电压采用100 KVp；管电流 < 40 mAs。总辐射暴露剂量≤5 mSv。(2)扫描范围从肺尖到肋膈角(包括全部肺)，患者吸气末一次屏气完成扫描。(3)扫描后原始数据行薄层重建，重建层厚为0.625 mm-1.25 mm。为方便进行计算机辅助检测及容积分析，建议层间有20%-30%重叠。(4)薄层重建算法建议采用软组织密度或肺算法，不建议采用高分辨率骨算法，引起对软件容积分析重复性影响较大。(5)肺结节的检测建议将薄层图像行三维重建，采用最大密度投影(maximal intensity projection, MIP)重建，有助于结节的检出及结节形态的观察。推荐应用计算机辅助检测(computer aided detection, CAD)软件结合人工阅片，提高结节检出率。

随访CT对肺结节生长性的评估方法包括肉眼评估，二维直径评估及三维体积评估。肉眼评估可以发现显著的结节生长，但对于较小结节及不显著的结节生长观察不准确，目前常规的评估方法为测量结节直径(最大层面长径与短径平均值)。计算机软件目前可以实现结节的容积测量(图 2)，其重复性优于直径测量。依据结节的直径或容积，计算容积倍增时间(volume doubling time, VDT)可作为量化结节生长速度的指标，其计算方法为：

**2:**
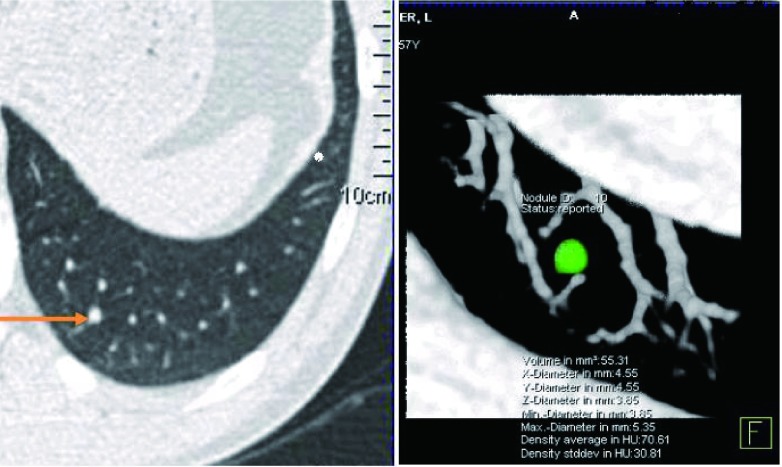
肺结节的容积测量 Volume measurement of lung nodules

VDTv(天)=[ln2^*^△t]/[ln(V2/V1)]

或VDTd(天) =[ln2^*^△t]/[3^*^ln(D2/D1)]

V代表体积，D代表直径，t代表两次扫描间隔时间

恶性实性结节的VDT多为30天-400天，而部分实性结节及磨玻璃密度结节常呈惰性生长，其容积倍增时间显著长于400天，因此需要长时间的CT随访^[16, 17]^。磨玻璃密度结节的生长不仅可以表现为体积的增长，也可以表现为CT值的增加或新出现实性成分，部分研究者引入质量测量(结节体积与密度乘积)，认为质量测量能更敏感的监测出非实性结节的生长变化^[17]^。

### 三 肺部结节恶性病变预测模型

目前有多种临床肺癌预测模型，其中以梅奥临床人员研发的模型应用最为广泛。此模型中包含6种预测肺恶性肿瘤的独立预测因素，包括年龄、吸烟(目前或曾吸烟)、结节发现前的胸腔恶性肿瘤史 > 5年、结节直径及毛刺、位于上叶^[18]^。预测模型的公式为：恶性概率=ex/(1+ex)(方程式1)，χ=-6.827, 2+(0.039, 1×年龄)+(0.791, 7×吸烟史)+(1.338, 8×恶性肿瘤)+(0.127, 4×直径)+(1.040, 7×毛刺征)+(0.783, 8×位置)(方程式2)。其中e是自然对数，年龄为患者的年龄(岁)，如果患者目前或者以前吸烟，则吸烟史=1(否则=0)；如果患者有胸腔外恶性肿瘤史 > 5年，则恶性肿瘤=1(否则=0)；直径为结节的直径(mm)，如果结节边缘有毛刺，则毛刺征=1(否则=0)；如果结节位于上叶，则位置=1(否则=0)。

## 3 肺部结节的肺癌风险评估处理策略

### 一 肺实性结节

(1) 肺癌高危结节

标准：直径≥15 mm或表现出恶性CT征像(分叶、毛刺、胸膜牵拉、含气细支气管征和小泡征、偏心厚壁空洞)的直径介于8 mm-15 mm之间的肺实性结节。

处理策略：肺癌高危结节均应由胸外科、肿瘤内科、呼吸科和影像医学科医师集体会诊，决定是否需要进行进一步检查(包括支气管镜、CT增强扫描、正电子发射(positron emission, PET)-CT扫描，经皮肺穿刺活检)明确诊断，以及采取什么方法进行治疗。对于高度怀疑为恶性者且适合于外科手术治疗者，首选外科治疗。对肺癌可能性较小的病例可抗炎治疗5天-7天，休息1个月后复查，结节增大或无变化者，由多学科会诊，决定是否进入临床治疗；结节缩小可在2年内进行随访(图 3)。

**3:**
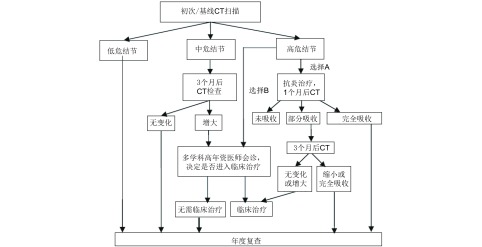
初次扫描实性肺结节处理流程 Treatment scheme of solid lung nodules by first scan

(2) 肺癌中危结节

标准：直径介于5 mm-15 mm且无明显恶性CT征象的非实性结节。

处理策略：应在3个月后进行随访观察其生长特性，发现结节生长纳入高危结节处理，无生长性则继续随访2年(图 4)。

**4:**
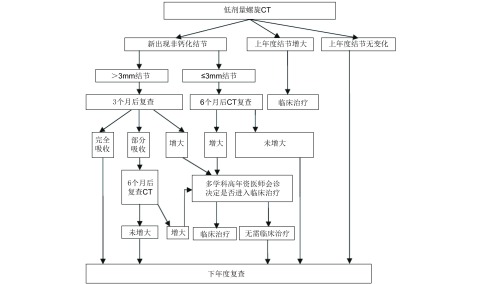
年度复查肺实性结节流程图 Treatment scheme of solid lung nodules by annual review scan

(3) 肺癌低危结节

标准：直径 < 5mm的实性结节

处理策略：肺癌低危结节建议1年后随访，发现生长则纳入高危结节处理，无生长行年度随访(图 3)。

(4) CT随访过程中的新发结节

根据其直径大小进行进一步处理，高危结节处理同基线扫描，鉴于新发结节的恶性可能性相对较大，其随访频率较基线扫描结节高(图 4)。

### 二 肺部分实性结节的风险评估及处理策略

鉴于部分实性结节的恶性概率在三种结节中最高，因此其肺癌风险度评价标准不同。

(1) 直径 > 8 mm的部分实性结节定义为高危结节，应由胸外科、肿瘤内科、呼吸科和影像医学科医师集体会诊，决定是否需要进行进一步检查(结节薄层三维重建CT扫描，薄层增强CT扫描，经皮肺穿刺活检)明确诊断、手术切除或3个月后进行CT复查。若结节3个月后没有缩小或增大时，考虑为恶性可能，建议手术切除。若结节缩小，建议6个月、12个月和24个月持续CT监测，无变化者建议长期年度CT复查，随访时间不小于3年。

(2) 直径≤8 mm的部分实性结节定义为中危结节，建议3个月、6个月、12个月和24个月持续薄层CT扫描，并作结节的薄层三维重建。如果结节具有生长性建议手术，无变化或缩小建议继续长期CT随访，随访时间不小于3年。

### 三 肺磨玻璃密度结节的风险评估及处理策略

(1) 直径 > 5 mm的纯磨玻璃密度结节定义为中危结节，建议3个月、6个月、12个月和24个月持续CT检测，结节具有生长性建议手术，无变化或缩小建议继续长期CT随访，随访时间不小于3年。

(2) 直径 < 5 mm的纯磨玻璃密度结节定义为低危结节，建议年度CT复查观察生长性。结节具有生长性建议手术，无变化或缩小建议继续长期CT随访，随访时间不小于3年。

### 四 多发肺结节的处理

多发肺结节的处理原则主要基于危险度最高的结节。对于多发高危险度结节，应考虑多原发肺癌的可能性，尤其是多发部分实性及亚实性结节。对此类结节，建议多学科会诊。

## 4 肺癌的治疗

对可疑肺癌的肺结节进行临床分期，检查方法包括支气管镜、胸部及全腹CT增强，头颅双倍剂量MRI增强，有条件可行PET-CT检查，对于确诊的肺癌依据类型和分期采用不同治疗方法。

### 一 非小细胞肺癌

(1)Ⅰ期肺癌治疗

包括Ⅰa期(T1a, bN0M0)和Ⅰb期(T2aN0M0)，治疗方法主要是手术切除，肺叶切除、纵隔淋巴结清扫以期实现R0切除是首选的治疗方式，完全切除的Ⅰa期患者不推荐辅助化疗。对不能耐受手术的患者，立体定向放疗(stereotactic ablative radiotherapy, SABR)可作为根治Ⅰ期肺癌的可行手段。此外，射频消融(radio frequency ablation, RFA)也是治疗选择之一，但RFA不推荐用于靠近肺部大血管的肿瘤。

(2)Ⅱ期肺癌治疗

Ⅱ期肺癌包括了Ⅱa(T1a-2aN1M0, T2bN0M0)期和ⅡB(T2bN1M0, T3N0M0)期，治疗方法与Ⅰ期肺癌一样，主要是手术治疗，手术切除后患者应常规行辅助化疗。

(3)Ⅲ肺癌的治疗

Ⅲ期肺癌包括Ⅲa和Ⅲb期，Ⅲ期肺癌为局部晚期非小细胞肺癌。从治疗观点看，可分为可切除和不可切除两大类。对可切除的局部晚期非小细胞肺癌，建议治疗方式为新辅助化疗+手术切除。对不可切除的局部晚期非小细胞肺癌标准的治疗模式为含铂方案化疗联合放射治疗。

(4)Ⅳ期肺癌的治疗

Ⅳ期肺癌的治疗以延长生命、提高生活质量为主要目的。

### 二 小细胞肺癌

早期(Ⅰ期和Ⅱ期)小细胞肺癌有外科手术治疗指征者，应施行外科治疗。Ⅲa期小细胞肺癌可以先行术前新辅助化疗，化疗后病变缓解，重新分期，定为Ⅱ期者可行外科治疗，Ⅲb期和Ⅳ期小细胞肺癌的治疗主要为化疗和放疗。

## 5 小结

与西方国家相比，我国肺癌发病的危险因素更为复杂，除吸烟外，严重的空气污染、生物燃料的使用以及女性非吸烟者中的肺癌发生，使得我国与这些国家的肺癌特征有所不同^[19, 20]^。因此在肺结节的处理中必须考虑中西肺癌特征的差异，基于此，我们结合国内外肺结节处理指南，结合我国LDCT肺癌筛查实践，制定了此指南。但目前仍有许多问题有待解决，如建立适合我国人群的肺癌临床预测模型，寻求可用于肺部结节良恶性判断的生物标志物等，都需要进一步的前瞻性临床研究来探索和验证。
