# 更正声明

**DOI:** 10.3779/j.issn.1009-3419.2023.103.02

**Published:** 2023-07-20

本刊2016年第19卷第12期刊登的题为“中国肺部结节分类、诊断与治疗指南（2016年版）” ［周清华, 范亚光, 王颖, 等. 中国肺部结节分类、诊断与治疗指南（2016年版）. 中国肺癌杂志, 2019, 19(12): 793-798.］一文中：第795页，（2）肺癌中危结节中“标准：直径介于5 mm-15 mm且无明显恶性CT征象的非实性结节”应改为“标准：直径介于5 mm-15 mm且无明显恶性CT征象的实性结节”。特此更正。对此深表歉意。

Erratum: China National Guideline of Classification, Diagnosis and Treatment for Lung Nodules (2016 Version)

Zhou QH, Fan YG, Wang Y, *et al*.

Zhongguo Fei Ai Za Zhi, 2016, 19(12): 793-798.

In the version of this article initially published, error appeared on page 795. The first sentence in part 3.1.2 should be “标准：直径介于5 mm-15 mm且无明显恶性CT征象的实性结节”.

